# Splenic CD11c(+) cells derived from semi-immune mice protect naïve mice against experimental cerebral malaria

**DOI:** 10.1186/s12936-014-0533-y

**Published:** 2015-01-28

**Authors:** Lam Q Bao, Dang M Nhi, Nguyen T Huy, Mihoko Kikuchi, Tetsuo Yanagi, Shinjiro Hamano, Kenji Hirayama

**Affiliations:** Department of Immunogenetics, Institute of Tropical Medicine, Nagasaki University, Nagasaki, Japan; Department of Clinical Product Development, Institute of Tropical Medicine, Nagasaki University, Nagasaki, Japan; Centre for International Collaborative Research, Nagasaki University, Nagasaki, Japan; Animal Research Centre for Tropical Infections, Institute of Tropical Medicine, Nagasaki University, Nagasaki, Japan; Department of Parasitology, Institute of Tropical Medicine, Nagasaki University, Nagasaki, Japan

**Keywords:** Semi-immune, CD11c(+) DCs, Plasmacytoid DCs, Cerebral malaria, Malaria-specific antibody

## Abstract

**Background:**

Immunity to malaria requires innate, adaptive immune responses and *Plasmodium*-specific memory cells. Previously, mice semi-immune to malaria was developed. Three cycles of infection and cure (‘three-cure’) were required to protect mice against *Plasmodium berghei* (ANKA strain) infection.

**Methods:**

C57BL/6 J mice underwent three cycles of *P. berghei* infection and drug-cure to become semi-immune. The spleens of infected semi-immune mice were collected for flow cytometry analysis. CD11c(+) cells of semi-immune mice were isolated and transferred into naïve mice which were subsequently challenged and followed up by survival and parasitaemia.

**Results:**

The percentages of splenic CD4(+) and CD11c(+) cells were increased in semi-immune mice on day 7 post-infection. The proportion and number of B220(+)CD11c(+)low cells (plasmacytoid dendritic cells, DCs) was higher in semi-immune, three-cure mice than in their naïve littermates on day 7 post-infection (2.6 vs 1.1% and 491,031 vs 149,699, respectively). In adoptive transfer experiment, three months after the third cured *P. berghei* infection, splenic CD11c(+) DCs of non-infected, semi-immune, three-cure mice slowed *Plasmodium* proliferation and decreased the death rate due to neurological pathology in recipient mice. In addition, anti-*P. berghei* IgG1 level was higher in mice transferred with CD11c(+) cells of semi-immune, three-cure mice than mice transferred with CD11c(+) cells of naïve counterparts.

**Conclusion:**

CD11c(+) cells of semi-immune mice protect against experimental cerebral malaria three months after the third cured malaria, potentially through protective plasmacytoid DCs and enhanced production of malaria-specific antibody.

## Background

Immunity to malaria in humans is a step-wise process that spans invasion of sporozoites into the body to development of erythrocytic parasites. This process involves humoral and cellular responses of innate immune cells (e.g., macrophages, dendritic cells (DCs)) and adaptive IFN-γ-producing CD4(+) and CD8(+) T cells [1]. Malaria infection at the individual and community levels is affected by acquired immunity. A person exposed to repeated *Plasmodium* infections may develop a partially protective immunity. Such ‘semi-immune’ persons often can be infected by malaria but rarely manifest the typical severe symptoms [2].

Memory immune cells are necessary to maintain immunity to microbial pathogens. The presence of *Plasmodium*-specific memory T and B cells in people living in malaria-endemic areas has been reported [3,4]. In a mouse model, memory CD4(+) T cells from chronically infected mice were shown to effectively delay and reduce parasitaemia and pathology [5]. In addition, memory CD8(+) T cells specific for *Plasmodium* liver-stage antigens prolong protection against malaria [4]. Coincidentally, both antibody and memory B cell responses to malarial antigens in people residing in regions with high *Plasmodium* transmission rates are stably maintained over time in the absence of re-infection [3,6,7].

CD11c(+) DCs are a major population of antigen-presenting cells that mediate interactions between the innate and adaptive immune responses and play an important role at the host-pathogen interface, including in responses to *Plasmodium* parasites [8-11]. Pattern recognition receptors expressed by DCs, such as toll-like receptors (TLRs), nod-like receptors, and C-type lectins, recognize distinct conserved microbial molecules [12]. DC maturation is critical in immunity to pathogenic micro-organisms because of the potential of these cells to stimulate differentiation of naïve CD4(+) T cells into various T helper (Th) cell types, including Th1, Th2, Th17, follicular Th cells, and induced regulatory T cells [13,14]. However, DC function can be compromised during the blood stage of malaria infection, as evidenced by the observation that *Plasmodium falciparum*-infected red blood cells adhere to human DCs via interaction between *P. falciparum* erythrocyte membrane protein 1 (PfEmP1) and CD36, thus inhibiting DC maturation and subsequently reducing the capacity of DCs to stimulate T cells [15,16]. However, the anti-malarial function of DCs in repeatedly malaria-infected hosts and the long-lasting protection of these cells remain unknown. This is the first study to show that CD11c(+) DCs from mice semi-immune to malaria contribute to the prolonged suppression of *Plasmodium* growth in the blood stage and prevent neurological pathology.

## Methods

### Animals and infection

C57BL/6 J (B6) mice aged six to eight weeks were supplied by SLC Laboratories, Fukuoka, Japan. Semi-immune mice were generated as described elsewhere [17,18]. Briefly, C57BL/6 mice were infected with 10^4^*Pb*A-pRBCs and then treated on day 5 after infection with intra-peritoneal injection of chloroquine (20 mg/kg/day) and pyrimethamine (20 mg/kg/day) daily for 7 days. Before subsequent rounds of infection, the parasite-clearance was confirmed and mice were rested for two weeks and then re-challenged with 10^4^*Pb*A-pRBCs. Mice that underwent three cycles of drug-cured infection became semi-immune. Challenge infections were performed by intraperitoneal injection of 10^5^*Plasmodium berghei* (ANKA strain) infected RBCs (iRBCs). *Plasmodium berghei* was selected for its capacity to induce experimental cerebral malaria (ECM) in B6 mice, with neurological signs (ataxia, paralysis, deviation of the head, and convulsions) appearing six to ten days after infection [19]. Three to four months after the third cured infection, semi-immune mice and age-matched controls were finally challenged with 10^5^*P. berghei*-iRBCs without subsequent treatment.

Approval from the local ethics committee for animal care and research was obtained, and all laboratory and animal practice guidelines of the Animal Centre of the Institute of Tropical Medicine (NEKKEN), Nagasaki, Japan, were adhered to. All experiments were conducted in accordance with local Animal Ethics Committee regulations (Ethical Review Committee at Institute of Tropical Medicine Nagasaki University).

### Flow cytometry

Spleen cells were collected and stained for cell surface antigens. Splenocytes were incubated on ice for 30 min with fluorochrome-conjugated mAbs against surface antigens. The mAbs used were anti-CD4 PE-cy7, CD8 FITC, CD19 Percp-cy5.5, CD11c APCs, CD11c APC-cy7, B220 PE-cy7, and isotype control Abs (BD Biosciences). Flow cytometric analysis was conducted using FACS Calibur, FACSCanto II, or FACSVerse systems (BD Biosciences), and the resulting data were analysed using Flow Jo software (Tree Star, Inc).

### Adoptive transfer

Three to four months after the third cured infection, non-infected, semi-immune and age-matched naïve B6 mice were sacrificed. Spleens were harvested and homogenized in collagenase D, and the resulting homogenates were resuspended in PBS, pH 7.2. After incubation with 25 μl of anti-CD11c microbeads (Miltenyi Biotec), 225 μl of cell suspension from one spleen was first sorted for positive selection. The purity of the CD11c(+) fraction was >95%. A total of 10^6^ CD11c(+) cells from either of semi-immune or naïve mice were intravenously adoptively transferred into six- to eight-weeks-old naïve B6 mice 24 hours before malaria infection with 10^5^*P. berghei*-iRBCs. Naïve control mice were injected with 200 μL of phosphate buffer saline (PBS). Parasitaemia was monitored daily by Giemsa-staining of thin blood smears from day 1 to day 8 post-infection and then every three to four days, and the degree of parasitaemia was expressed as a percentage determined from examination of more than 1,000 RBCs. Mice in each group were monitored daily for symptoms of ECM, such as coma, convulsions, lethargy, tremors, ataxia, hemiplegia, and paraplegia.

### ELISA

Plasma harvesting and measurement of plasma *P. berghei*-specific IgG subtypes were conducted as described in a previous study [17]. The entire set of experiments was performed at least twice.

## Results

This is the first study demonstrating that splenic CD11c(+) cells from semi-immune mice are capable of prolonged inhibition of malaria parasite development and prevention of ECM. This semi-immune mouse model was developed by repeated infection and radical anti-malaria treatment, mimicking the natural boost infection and recovery in humans in areas of intense *P. falciparum* transmission. In previous work, it was shown that mice that undergo three cycles of infection and cure retain resistance to ECM 12 months after the third exposure to malaria.

### A history of malaria exposure increased the population of CD4(+), CD11c(+), B220(+)CD11c(+)low cells and the size of CD11c(+)hi cells in semi-immune mice on day 7 post-infection

**Figure 1:**
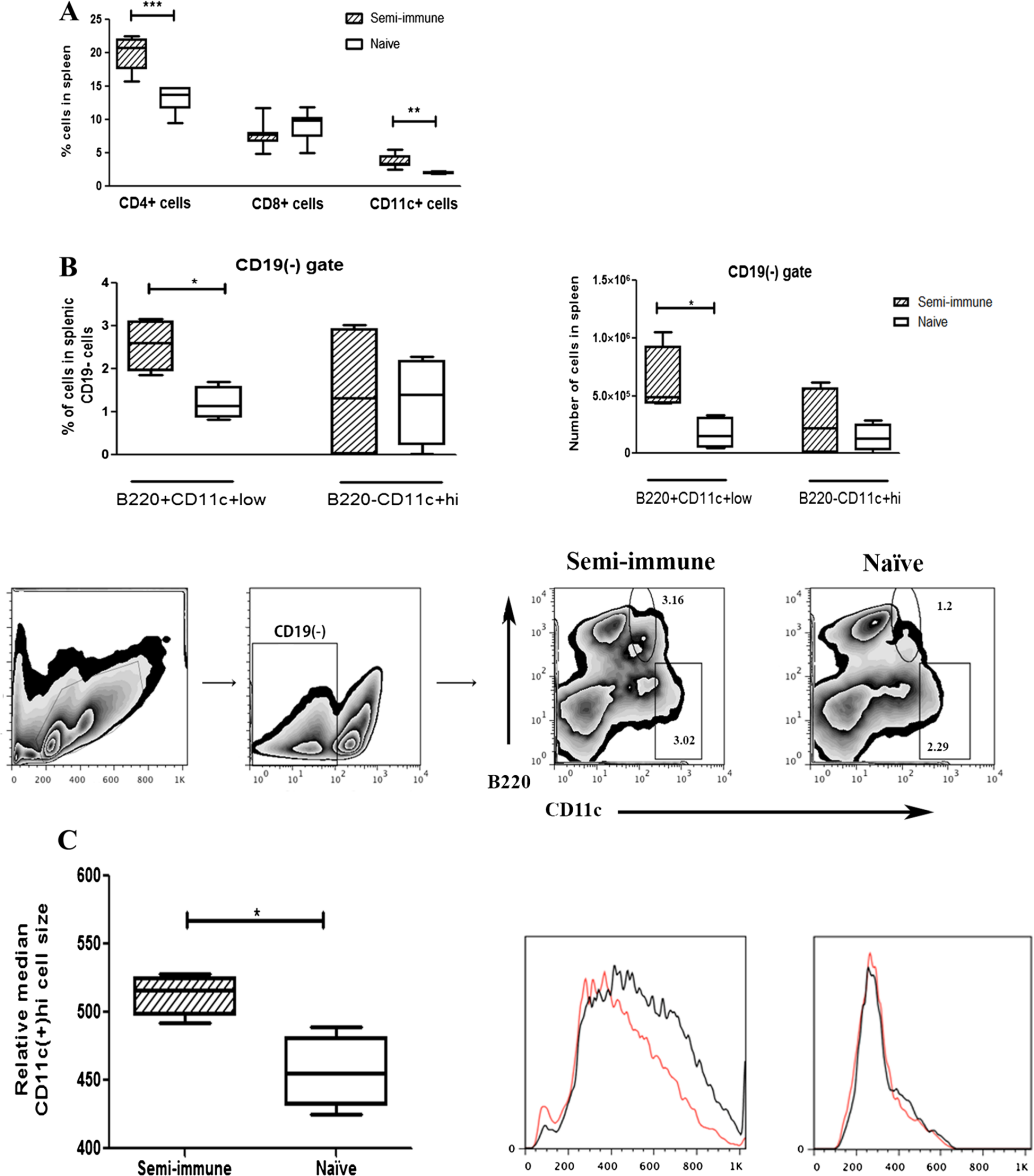
**The proportion of B220(+)CD11c(+)low cells was higher and the size of CD11c(+)hi cells was enhanced on day 7 post-infection in semi-immune mice challenged three months after the third exposure to malaria. (A)** Median percentages of splenic CD4(+), CD8(+) and CD11c(+) cells on day 7 post-infection (±interquartile range) (n = 6-7 mice/group). **(B)** Median percentages and number of splenic B220(+)CD11c(+)low cells and B220(−)CD11c(+)hi cells on day 7 post-infection (±interquartile range) (n = 4 mice/group). Plots show the gating strategy and data for representatives of these cells on day 7 post-infection in semi-immune and naïve mice. **(C)** Relative median size of splenic CD11c(+)hi cells on day 7 post-infection (±interquartile range) (n = 4 mice/group). Representative histograms regarding the size of CD11c(+)hi cells in semi-immune (black) and naïve (red) mice on day 0 and day 7 post-infection are shown. Data are pooled from two independent experiments **(A, B)** or representative of two independent experiments **(C)**. *P < 0.05, Mann–Whitney *U* test.

It was found that the percentage of splenic CD4(+) and CD11c(+) cells in semi-immune mice on day 7 post-infection was significantly higher than in infected naïve counterparts (Figure 1A), suggesting a potentially protective cooperation of CD4(+) and CD11c(+) cells against *Plasmodium* infection. Meanwhile, no significant difference in proportion of CD8(+) cells was observed between these two groups of mice (Figure 1A). Thus, this study was carried out with the hypothesis that CD11c(+) cells from semi-immune mice contributed to immunity against ECM. In murine malaria infection, splenic CD11c(+) cells are considered as splenic CD11c(+) DCs [20]. The role of DCs in prolonged suppression against malaria is still unclear. Hence, the anti-malaria capacity of CD11c(+) DCs and their subtypes was investigated from semi-immune mice in long term after the last immunization. B220(+)CD11c(+)low cells, named as plasmacytoid DCs [21,22], play a critical role in immunity to malaria [21] and in improving the function of conventional DCs [23]. The distribution of this DC subtype in the spleen of semi-immune mice was, therefore, examined. Three months after the third cured malaria infection, B220(+)CD11c(+)low cells in CD19(−) gate accounted for a significantly higher percentage and showed a greater number of cells on day 7 post-infection in infected semi-immune mice than in naïve littermates (2.6 and 1.1%; 491,031 and 149,699, respectively; *P* < 0.05) (Figure 1B). Meanwhile, no differences in the proportion or number of B220(−)CD11c(+)hi cells (conventional DCs) was observed between these two groups (1.3 and 1.4%; 222,096 and 127,144, respectively) (Figure 1B). The increase in the proportion of plasmacytoid DCs in semi-immune mice probably enhanced the activation of conventional DCs and, therefore, stimulated the proliferation of T cells, leading to parasite clearance.

The precise role of plasmacytoid DCs remains unclear. There is, however, growing evidence that the role of plasmacytoid DCs is to support and improve the function of conventional DCs [23]. In non-lethal *Plasmodium chabaudi* infections, the numbers of both splenic plasmacytoid and conventional DCs are increased, but only conventional DCs upregulate the expression of co-stimulatory molecules and present *Plasmodium* antigens, resulting in activation of CD4(+) T cells [24,25]. A recent mouse model study showed that up to 70% of plasmacytoid DCs in the spleen had been in contact with *Plasmodium* spp. and that a small percentage of these cells were reservoirs of infectious parasites [20]. Significantly, interaction between CD40 on conventional DCs and the CD40 ligand on plasmacytoid DCs leads to IL-12 production by conventional DCs, inducing immune activation [26]. The function of plasmacytoid DCs in malaria should be confirmed in a future study.

In addition, it was found that CD11c(+)hi cells (conventional DCs) were larger in size on day 7 post-infection in semi-immune mice than naïve counterparts, with forward scatter relative median sizes of 517 and 456, respectively (*P* < 0.05) (Figure 1C). There was no difference in the size of CD11c(+)hi cells on day 0 in these two groups of mice (Figure 1C). The increased size of conventional CD11c(+)hi DCs in the semi-immune mice probably played a role in the beneficial effects of these DCs in the host’s immune response to the parasites, as increases in the size of immune cells during malaria infection have been reported. For example, the size and granularity of splenic DCs were shown to increase in mice with malaria [27]. Immature DCs are much smaller in size than mature DCs [28]; therefore, the increased size of CD11c(+)hi conventional DCs observed in this study could have functional relevance for immunity against malaria.

### Naïve B6 mice receiving splenic CD11c(+) cells from donor semi-immune mice exhibited lower parasitaemia and were partially protected from development of ECM during the acute phase of malaria infection

**Figure 2:**
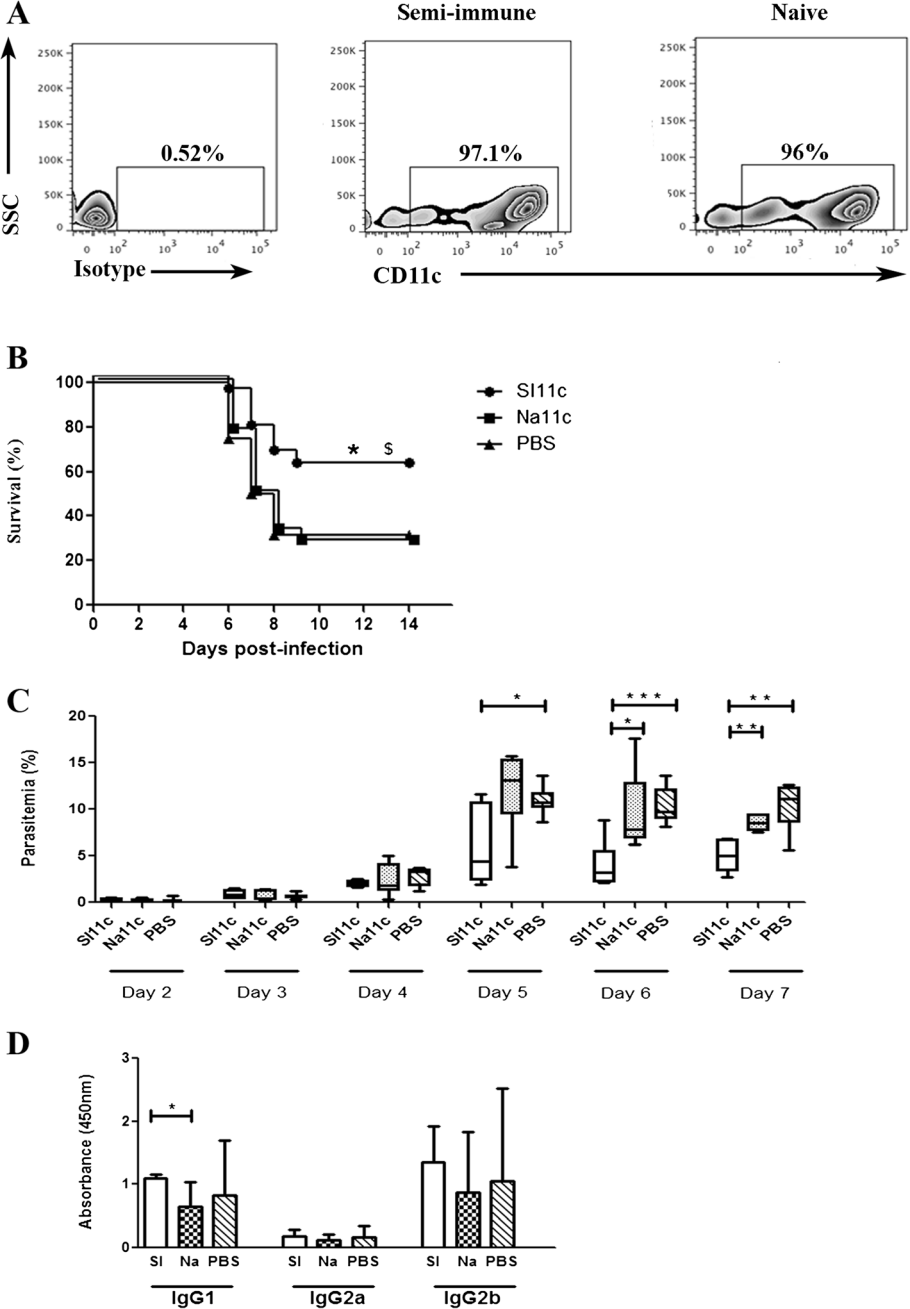
**Splenic CD11c(+) cells from semi-immune mice lowered parasitaemia, enhanced anti-** ***P. berghei*** **IgG1 level and improved the ECM survival rate in recipient mice. (A)** Plots show the representative purity of CD11c(+) cells. **(B)** Survival curves for mice that received 10^6^ splenic CD11c(+) cells and for PBS-treated control mice (n = 16-18 mice/group). Data shown are pooled from three independent experiments. Significant differences between groups as determined by log-rank test are indicated by symbols: *P < 0.05 for SI11c *vs* Na11c; $*P* < 0.05 for SI11c *vs* PBS. **(C)** Bar chart showing the median parasitaemia (%) of recipient mice over the time of infection (n = 6-9 mice/group). Parasitaemia (%) of these groups of mice was performed on days post challenge as indicated and was shown by the box-plots. Data shown are representative of three independent experiments. *P < 0.05; **P < 0.01; ***P < 0.001, Mann–Whitney *U* test. **(D)** Median levels of plasma anti-*P. berghei* IgG1, IgG2a and IgG2b were determined on day 5 post-infection in mice with adoptively transferred DCs. Data are representative of two separate experiments (n = 4 mice/group). *P < 0.05, Mann–Whitney *U* test.

To examine the long-lasting anti-malaria role of splenic CD11c(+) DCs derived from semi-immune mice, CD11c(+) cells was isolated with the purity >95% (Figure 2A) from the spleens of semi-immune mice three to four months after the third cured infection, as well as from age-matched naïve controls and passively transferred these cells to eight-weeks-old naïve mice. As shown in Figure 2B, 61% of mice receiving CD11c(+) cells from semi-immune mice (SI11c) were resistant to ECM and survived for 14 days post-infection. Finally, all of these mice succumbed to severe anemia and high parasitaemia around days 28–30 post-infection. Conversely, the survival rates of PBS-treated control mice and mice that received CD11c(+) cells from age-matched naïve mice (Na11c) were significantly lower during the first two weeks of infection, at 28 and 31%, respectively (*P* <0.05). Splenic CD11c(+) DCs from semi-immune mice with last immunization three months previously partially protected naïve B6 mice against ECM in the passive transfer experiments, confirming the beneficial effects of CD11c(+) DCs in prolonged protection against pathology. CD11c(+) cells from semi-immune mice had a noticeable attenuating effect on parasitaemia in recipients beginning on day 5 post-infection compared with PBS-treated control mice and those that received CD11c(+) cells from naïve mice (Figure 2C). It is known that in malaria infection, DCs containing RBCs expressed higher type I interferon[IFN] levels than those without RBCs [21]. Donor CD11c(+) DCs from semi-immune mice, including RBC containing DCs, probably extendedly produced more type I IFN than naïve DCs and then suppressed the parasite proliferation. Consequently, DCs could be one of effective cells for low parasitaemia which was previously observed in semi-immune mice [17]. Prevention of ECM through a lowering of parasitaemia correlated with reduced levels of pathogenic cytokines, chemokines, CD8(+) T cells, and parasites in the brain has been reported [29-31]. Hence, in the present study, the decrease in parasitaemia mediated by semi-immune DCs probably contributed to the improved survival rate of recipient mice during the acute phase of malaria. CD11c(+) DCs, major innate immune cells, are important in the development of immunity to malaria. Previous studies in mice have demonstrated that in malarial infections that prove lethal, the function of DCs is compromised, but functional DCs improve the survival rate in non-lethal malaria [23,32-35].

### Donor CD11c(+) cells from semi-immune mice induced anti-*P. berghei* IgG1 antibody production in recipient mice during the acute stage of infection

To identify the type of immune response provoked in mice receiving transferred cells, the levels of plasma IgG subclasses specific to malaria were measured. The level of helper type 2 T cell (Th2)-mediated anti-*P. berghei* IgG1 production [36] in semi-immune CD11c(+) cell-treated mice was significantly enhanced compared with naïve CD11c(+) cell-treated mice (*P* < 0.05). Similar production of helper type 1 T cell (Th1)-mediated *Plasmodium*-specific IgG2a and IgG2b [36] was observed in all three mouse groups (Figure 2D). Although the difference was not significant, the *P. berghei*-specific IgG1 level was higher in mice that received semi-immune CD11c(+) cells than in PBS-treated controls (Figure 2D). These data indicate that Th2 activation via IL-4, Il-5 or IL-13 could be associated with downstream signalling by protective semi-immune DCs. The signalling pathway of semi-immune DCs should be studied further in the future.

## Conclusion

Protective CD11c(+) DCs from malaria-exposed mice survive for extended periods, despite the absence of further exposure to malaria. These cells prevent development of ECM and lower the degree of parasitaemia. Full elucidation of the role of *Plasmodium*-exposed CD11c(+) DCs and plasmacytoid DCs in malaria and other infectious diseases requires further study. The findings of the present study may provide the background necessary to prolong the protective immunogenicity of potential vaccine candidates.
